# End-to-End Fusion of Hyperspectral and Chlorophyll Fluorescence Imaging to Identify Rice Stresses

**DOI:** 10.34133/2022/9851096

**Published:** 2022-08-02

**Authors:** Chu Zhang, Lei Zhou, Qinlin Xiao, Xiulin Bai, Baohua Wu, Na Wu, Yiying Zhao, Junmin Wang, Lei Feng

**Affiliations:** ^1^School of Information Engineering, Huzhou University, Huzhou 313000, China; ^2^College of Mechanical and Electronic Engineering, Nanjing Forestry University, Nanjing, China; ^3^College of Biosystems Engineering and Food Science, Zhejiang University, Hangzhou 310058, China; ^4^Key Laboratory of Spectroscopy Sensing, Ministry of Agriculture and Rural Affairs, Hangzhou 310058, China; ^5^Institute of Digital Agriculture, Zhejiang Academy of Agricultural Sciences, Hangzhou 310021, China; ^6^Institute of Crop Science and Nuclear Technology Utilization, Zhejiang Academy of Agricultural Sciences, Hangzhou 310021, China

## Abstract

Herbicides and heavy metals are hazardous substances of environmental pollution, resulting in plant stress and harming humans and animals. Identification of stress types can help trace stress sources, manage plant growth, and improve stress-resistant breeding. In this research, hyperspectral imaging (HSI) and chlorophyll fluorescence imaging (Chl-FI) were adopted to identify the rice plants under two types of herbicide stresses (butachlor (DCA) and quinclorac (ELK)) and two types of heavy metal stresses (cadmium (Cd) and copper (Cu)). Visible/near-infrared spectra of leaves (L-VIS/NIR) and stems (S-VIS/NIR) extracted from HSI and chlorophyll fluorescence kinetic curves of leaves (L-Chl-FKC) and stems (S-Chl-FKC) extracted from Chl-FI were fused to establish the models to detect the stress of the hazardous substances. Novel end-to-end deep fusion models were proposed for low-level, middle-level, and high-level information fusion to improve identification accuracy. Results showed that the high-level fusion-based convolutional neural network (CNN) models reached the highest detection accuracy (97.7%), outperforming the models using a single data source (<94.7%). Furthermore, the proposed end-to-end deep fusion models required a much simpler training procedure than the conventional two-stage deep learning fusion. This research provided an efficient alternative for plant stress phenotyping, including identifying plant stresses caused by hazardous substances of environmental pollution.

## 1. Introduction

Rice (*Oryza sativa* L.) is one of the main staple food in the world. With the development of breeding techniques, high yield and stress-resistant varieties have been developed and promoted for planting to increase the rice yield. However, the yield will reach the plateaus when the planting fields no longer increase. On the other hand, climate instability and biotic and abiotic stresses show great threats to rice production. Although stress-resistant varieties have been bred, they can only deal with a few stresses. These varieties also suffer from a complex growth environment, resulting in yield and quality loss.

Herbicides and heavy metals are the main hazardous substances causing environmental pollution, resulting in the abiotic stress of plants. Generally, the rice plants are exposed to herbicides or heavy metals, which might not cause death to the plants, and the plants under the threat of relatively low concentrations of herbicides and heavy metals might not show obvious differences in the symptoms. Under this situation, it is hard to identify the stress types of plants. Moreover, the stresses will affect plant growth, and different plant organs may respond differently. The acquisition of phenotyping traits of different organs can provide complementary information of plants, improving the precision and stability of plant growth status monitoring. Accurately and automatically identifying abiotic stress types can obtain plant growth information and find the optimal solution to deal with the stresses. Knowing what happens to the plants and how to treat them makes it possible to minimize the influence of abiotic stresses to stabilize the yield and quality.

High-throughput phenotyping of plants can help obtain a large number of phenotyping traits in digital and automatic manners. The development of modern analytical techniques has made great contributions to high-throughput plant phenotyping [1–5]. The numerous data acquired by the analytical instruments contained various information relating to plant growth status. The plant phenotyping traits can evaluate the plant growth status. High-throughput phenotyping also helps study the relationship between phenomics and genomics [6, 7]. Hyperspectral imaging (HSI) [8–11] and chlorophyll fluorescence imaging (Chl-FI) [11–13] are two widely used techniques for high-throughput plant phenotyping, providing different information on plant growth. HSI and Chl-FI have been studied for heavy metal and herbicide stresses [14–17].

HSI integrates the spectroscopy technique and imaging technique, capturing spectral information of pixels within the image. The spectral information is related to the chemical compositions and physiological and biochemical reactions. Image information relates to external information, such as plant structure, color, and morphological features. Full-range spectra, feature wavelengths, and spectral indices derived from full-range spectra are mostly used for analysis. Chl-FI has also been used for high-throughput phenotyping of plants. It captures chlorophyll fluorescence signals from the samples, which relates to photosynthesis. Chlorophyll fluorescence kinetic parameters are mostly used for analysis, and some studies have used chlorophyll fluorescence spectra for analysis [18–20].

Previous studies have proved the effectiveness of HSI and Chl-FI for plant stress phenotyping individually. The combination of HSI and Chl-FI has also been studied for plant stress phenotyping [21–23]. However, most of these studies have analyzed HSI and Chl-FI separately [21–24]. Since HSI and Chl-FI acquired different phenotyping traits based on different principles, the fusion of the features of HSI and Chl-FI for plant stress type identification can be explored to use complementary information of different phenotyping traits. Information fusion has been widely used for integrating multimodal or multisensor data to improve analysis performances for different purposes with higher precision and reliability. In the review of [25], the authors discussed the potential of information fusion of HSI and Chl-FI. In the review of [26], the authors also discussed the potential of information fusion for plant stress phenotyping. Various studies have shown the good performances of information fusion for plant phenotyping, including the fusion of data acquainted by different types of sensors (representing different techniques) [27–35].

Deep learning has been the hottest topic in machine learning and artificial intelligence. With the ability to learn deep and representative features from big data, deep learning has been used in various fields, including plant phenotyping. Deep learning has also been used for high-throughput plant phenotyping [26, 36–40]. According to previous studies, shallow CNN models can work well on one-dimensional (1D) spectral data [14, 41]. To our knowledge, no previous studies have used the Chl-FKC as inputs of CNN. The 1D Chl-FKC is similar to the VIS/NIR spectra. Due to the significant feature learning ability, DL can fuse features from VIS/NIR spectra and Chl-FKC to fully reveal the phenotyping information of plants. Researchers have conducted deep learning-based information fusion for plant phenotyping [38, 42, 43].

In general, there are three different levels of information fusion, including low-level fusion (fusion of original data), middle-level fusion (fusion of features extracted by a feature extractor), and high-level fusion (decision fusion) [44–46]. However, most existing information fusion models are built based on a two-stage training procedure, requiring individual feature extractors and classifiers. The first step is to train a feature extractor or directly use a manually defined one to produce the features for fusion. The second step is to train another model for discrimination based on the fused features obtained in the first step [27, 28, 33]. These information fusion models are complex and need manual intervention. Recent studies have developed end-to-end deep fusion models for applications, combining feature extraction and modeling in one model [47, 48]. The features are automatically learned, fused, and fed into the classifier. The end-to-end deep fusion models have simpler training procedures and are more applicable for real-world applications.

This study focused on identifying different types of abiotic stresses using HSI and Chl-FI techniques. The specific objectives were to (1) identify two types of herbicide stresses (butachlor (DCA) and quinclorac (ELK)) and two types of heavy metal stresses (cadmium (Cd) and copper (Cu)), (2) explore the performances of the stress type identification using different techniques (HSI and Chl-FI) and different organs (leaves and stems), and (3) explore the stress type identification using the three levels of end-to-end deep fusion to fuse the information acquired by different techniques (HSI and Chl-FI) and different organs (leaves and stems).

## 2. Materials and Methods

### 2.1. Sample Preparation

The rice variety used in this study was Zhongheyou 4, provided by the Institute of Crop Science and Nuclear Technology Utilization, Zhejiang Academy of Agricultural Sciences. The rice seeds were sowed onto the seedbed. The rice seedlings were transplanted to the laboratory for stress one month later. The rice seedlings were transplanted in plug trays with nutrient soil. Regular water management and fertilizer management were conducted. After one week of transplantation, the seedlings were used for treatments with different stresses.

Two different herbicides were used, including butachlor (DCA) and quinclorac (ELK). Two heavy metals were used, including copper (Cu) and cadmium (Cd). For butachlor stress, butachlor solutions with a 50% active constituent were purchased from the local pesticide shop, and the dosage of used butachlor solutions was 0 (control (CK)), 1.5, 3, and 6 mL/L. For quinclorac stress, quinclorac powders with a 99% active constituent were purchased, and the dosage of used quinclorac powder was 0 (CK), 0.56, 1.12, and 2.24 g/L. For Cu and Cd stresses, the concentration of Cu and Cd was 0 (CK), 10, 30, and 60 *μ*mL/L. The different concentrations of herbicides and heavy metals were used to include more sample variations under one type of stress. In all, the number of samples under the treatments of CK, Cd, Cu, DCA, and ELK was 240, 360, 360, 268, and 358, respectively.

After the first time of stress, regular water management and fertilizer management were conducted. The efficiency of hyperspectral image acquisition and chlorophyll fluorescence image acquisition makes it unable to acquire a lot of samples a day. Thus, the four stresses were conducted on four successive days. After one week, the image acquisition of the corresponding stresses was conducted on another 4 successive days for the second batch of samples. As for hyperspectral image acquisition and chlorophyll fluorescence image acquisition, both leaves and stems were cut from the seedling and used for image acquisition separately.

### 2.2. Hyperspectral Image Acquisition

An assembled hyperspectral imaging system (as described previously in [49]) was used to acquire hyperspectral images of rice leaves and stems. The system covers the spectral range from 380 to 1030 nm, integrated with a spectrograph, a camera with a lens, and a tungsten halogen light source. The samples could be placed on a conveyer belt driven by a stepper motor. The hyperspectral images were collected using line-scan mode and calibrated by black-white calibration.

(1) $$\begin{array}{c} I=\frac{I_{\text{raw}}-I_{\text{black}}}{I_{\text{white}}-I_{\text{black}}}, \end{array}$$

where *I* denotes the calibrated spectral image. *I*_raw_, *I*_black_, and *I*_white_ denote the raw image, black image, and white image.

The system parameters were adjusted to acquire clear and nondeformable images. The camera exposure time was set as 0.027 s, the speed of the moving plate was set as 3.4 mm/s, and the distance between the lens and the moving plate was adjusted to 26 cm. It was expected to cover the tested plant as much as possible, and the plant was cut into segments and put symmetrically under the camera (on the conveyor belt) for line-scan image collection.

### 2.3. Chlorophyll Fluorescence Image Acquisition

A pulse-amplitude-modulated chlorophyll fluorescence imaging system (FluorCam FC800, Photon Systems Instruments, Brno, Czechia) (as described previously in [50]) was used to acquire chlorophyll fluorescence images. This chlorophyll fluorescence imaging system consists of a CCD camera (1392 × 1040 resolution) with an industry lens SV-H1.4/6 (VS Technology, Tokyo, Japan). The light source is formed by five light-emitting diodes (LED). An elevating table (HTVS120, SPL, Hangzhou, China) is used to adjust the distance between the samples and the lens. The image acquisition procedure was the same as [51], while the system parameters differed. When acquiring the chlorophyll fluorescence images, the actinic lights, saturating flashes, exposure time, and sensitivity were adjusted at 90%, 75%, 33.3 *μ*s, and 33.3%. The segmented plants were placed inside a dark room (the area that the camera could cover) for chlorophyll fluorescence image acquisition.

### 2.4. Spectral Data Extraction and Chlorophyll Fluorescence Kinetic Curve Extraction

#### 2.4.1. Spectral Curve Extraction

For hyperspectral images, the leaves of a sample are identified as the region of interest (ROI) of leaves (LROI), and the stem of a sample is also identified as an ROI (SROI). Considering the fact that the head and the end of the spectra contained obvious noises caused by the instrument, only the visible/near-infrared (VIS/NIR) spectra in the range of 454-957 nm (396 wavebands) were used for analysis. The pixel-wise spectra within the ROI were preprocessed by wavelet transform (wavelet function Daubechies 8 with decomposition level 3). All pixel-wise spectra within each ROI were averaged as the spectrum of the samples. The VIS/NIR spectra of leaves (L-VIS/NIR) and stems (S-VIS/NIR) were obtained for each rice plant, respectively. For chlorophyll fluorescence images, the leaves and the stem were identified as ROIs (similar to the ROI definition in hyperspectral images), respectively. Pixel-wise Chl-FKC within each ROI were averaged as the chlorophyll fluorescence kinetic spectrum. The Chl-FKC of leaves (L-Chl-FKC) and stems (S-Chl-FKC) were obtained for each rice plant, respectively. Maximum normalization was conducted to preprocess the VIS/NIR spectra and the Chl-FKC for further modeling procedures.

#### 2.4.2. Dataset Preparation

To establish models, the class labels of the samples in the control group (CK) and the class labels of the samples under the stresses of Cd, Cu, DCA, and ELK were assigned as 0, 1, 2, 3, and 4, respectively. A typical dataset split approach in the deep learning area was adopted. For each group, the samples were randomly split into the training, validation, and testing sets [52–54], using the ratio of 4 : 1 : 1. The samples in the training, validation, and testing sets were the same for each single data source. The details of the dataset are listed in Table 1.

### 2.5. Classification Models

#### 2.5.1. Conventional Machine Learning Methods

The support vector machine (SVM) is a widely used pattern recognition method [55]. For linearly separable issues, a linear classier is developed. For nonlinear classification, SVM maps the original data into high dimensions by kernel functions and establishes hyperplanes to maximally classify the closest training samples of different classes. In this study, SVM was used to compare deep learning approaches.

#### 2.5.2. Deep Learning Method

The convolutional neural network (CNN) is a widely used deep learning algorithm. The extracted VIS/NIR spectra and Chl-FKC were used to identify the stress types in this study.

**Figure 1:**
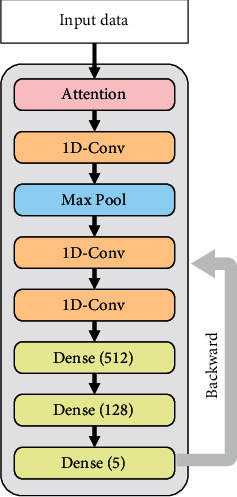
The architecture of the CNN model with a single type of input. “1D-Conv” means 1D convolution, and “Max Pool” denotes the max-pooling layer.

A 1D CNN architecture was designed as the base classifier for processing each single data source. This model was defined as CNN-S.  Figure 1 shows the shallow CNN architectures used in this study. The first part was an attention layer, which operated based on

(2) $$\begin{array}{c} Y_{\text{attention}}=\theta_{\text{RELU}}\left(W_{1}\cdot \theta_{\text{RELU}}\left(W_{2}\cdot X+b_{1}\right)+b_{2}\right)\cdot X, \end{array}$$

where *W*_1_, *W*_2_, *b*_1_, and *b*_1_ are the trainable parameters (weights and bias) in the attention layer, *θ*_RELU_ is the activation function, and *X* denotes the input. The second part was a 1D convolution block consisting of three 1D convolution layers (kernel size of 3, stride of 1; activated using RELU). To reduce the dimension of features, a max-pooling layer (pool size of 2, stride of 2) was added after the first convolution layer. The third part was a fully connected neural network including three dense layers. The numbers of neurons were 512, 128, and 5, respectively, using RELU activation. Furthermore, a batch normalization layer was added before each convolution and dense layer. The SoftMax Cross-Entropy was utilized as the loss function.

### 2.6. Fusion Strategies

**Figure 2:**
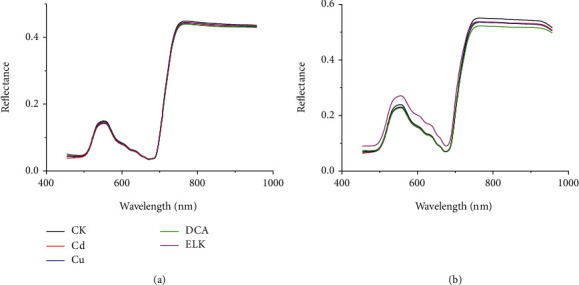
Average VIS/NIR spectra of the leaves (a). Average VIS/NIR spectra of the stems (b).

**Figure 3:**
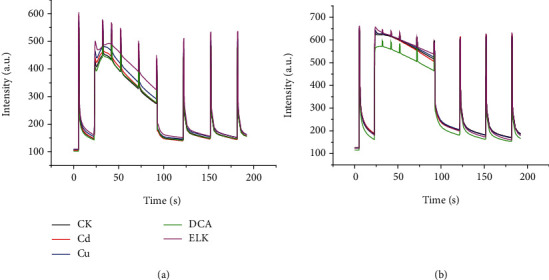
Average Chl-FKC of the leaves (a). Average Chl-FKC of the stems (b).

Leaves and stems showed different physiochemical characteristics under stress. During the seedling stage of rice, the leaves and stems are all green, and the VIS/NIR spectra and Chl-FKC of leaves and stems are quite similar (Figures 2 and 3). Since HSI and Chl-FI acquired different phenotyping traits based on different principles, each technique can provide limited plant phenotyping information. Thus, the fact that the fusion of HSI and Chl-FI can help identify rice stress types is worthy of investigation. There were two organs (leaves and stems) and two techniques (HSI and Chl-FI). Five different fusion strategies of data were conducted according to Table 2. It should be noted that L-VIS/NIR, S-VIS/NIR, L-Chl-FKC, and S-Chl-FKC of the same sample were used for fusion.

This study explored three different levels of information fusion (low level, middle level, and high level). For each information fusion level, end-to-end deep fusion models were developed. For low-level fusion, the original data of each type of feature were concatenated directly as a long vector and performed the classification task by the CNN-S model according to the fusion strategies in Table 2.

**Figure 4:**
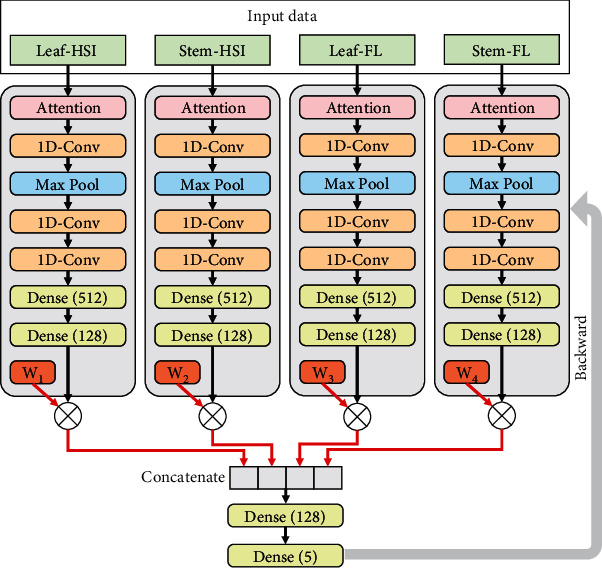
The architecture of the end-to-end CNN model for middle-level fusion using all the four types of features. HSI: hyperspectral imaging; FL: fluorescence; 1D-Conv: 1D convolution. *W*_1_ to *W*_4_ were the weights for fusion.

For middle-level fusion, an end-to-end CNN architecture for information fusion was proposed to fuse different types of original spectra for plant stress type identification. Each type of original feature was fed into the corresponding feature extractor, and the model required only a one-stage training. The fusion strategies for the end-to-end CNN were based on Table 2. The middle-level fusion model fused the deep features for further classification. In Figure 4, the end-to-end CNN model for middle-level fusion using all the four types of features is presented. Four single CNN-S models were employed for processing different data sources. In particular, the last dense layer of the CNN-S was removed. Therefore, the 128-dimension output (4 vectors from the second dense layer of each CNN-S submodel) was considered the deep features. They were weighted and concatenated for fusion.

The middle-level fusion could be described using

(3) $$\begin{array}{c} Y_{\text{mid}}=\varphi_{\text{Concat}}\left(\theta_{\text{RELU}}\left(W_{1}\right)F_{1},\theta_{\text{RELU}}\left(W_{2}\right)F_{2},\theta_{\text{RELU}}\left(W_{3}\right)F_{3},\theta_{\text{RELU}}\left(W_{4}\right)F_{4}\right), \end{array}$$

where *φ*_Concat_ means that this function concatenates all inputs to generate a vector; *F*_1_, *F*_2_, *F*_3_, and *F*_4_ are the deep features from L-VIS/NIR, S-VIS/NIR, L-Chl-FKC, and S-Chl-FKC, respectively.

**Figure 5:**
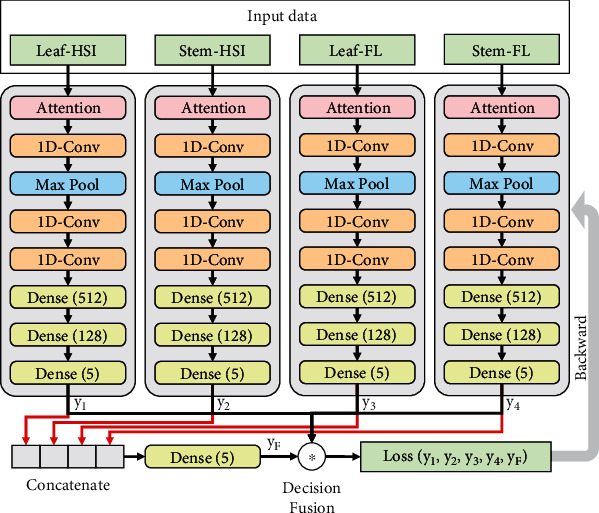
The architecture of the end-to-end CNN model for end-to-end high-level fusion using all the four types of features. HSI: hyperspectral imaging; FL: fluorescence; 1D-Conv: 1D convolution. *y*_1_ to *y*_4_ were the outputs of the single model.

For high-level fusion, another end-to-end CNN model was designed (shown in Figure 5). In this paper, high-level fusion could also be understood as decision fusion, which provides the final decision on classification based on the output of different classifiers. Different from the mentioned middle-level fusion model, the high-level fusion model used four complete CNN-S models to process the four data sources. Then, the outputs of the four submodels (four vectors with a shape of 5∗1) were concatenated and further fed into another dense layer with five neurons. It should be pointed out that a new loss function was proposed especially for this deep decision fusion model; see

(4) $$\begin{array}{c} \text{Loss}=\ell \left(y,y_{\text{F}}\right)+\ell \left(y,y_{1}\right)+\ell \left(y,y_{2}\right)+\ell \left(y,y_{3}\right)+\ell \left(y,y_{4}\right), \end{array}$$

where *y*_F_ denotes the output of the whole decision fusion-based deep learning model; *y*_1_, *y*_2_, *y*_3_, and *y*_4_ are the outputs of four submodels; and *ℓ* denotes the Cross-Entropy loss. This loss function could simultaneously promote the final classification loss and that of the submodels as small as possible. Moreover, due to the constraint of the loss function on the output of submodels, the fused *y*_1_, *y*_2_, *y*_3_, and *y*_4_ were forced to approach the expected probability distribution. Thus, the combination of them was a typical decision fusion.

To evaluate the superiority of the proposed methods, two-stage middle- and high-level fusion approaches were also used for comparison. In this study, only the two-stage fusion of all the four types of features was implemented. As for middle-level fusion, feature extraction, feature fusion, and modeling were conducted separately. The features were learned and extracted from CNN-S models using each single type of feature, and all the features extracted from the four types of features were concatenated. The fused features were then fed into a CNN-S model. As for high-level fusion, the predicted probability distribution vectors of the CNN-S models using four data sources were first extracted. Then, the averaged probability distribution vectors were used to make the final decision.

### 2.7. Model Performance Evaluation and Software

The classification accuracy was evaluated by the ratio of the number of correctly classified samples to the number of total samples. SVM was conducted on scikit-learn (version: 1.0.1) in Python 3.7, and CNN was conducted using the MXNet framework (Amazon, Seattle, WA, United States) with Python 3.7.

## 3. Results

### 3.1. Profiles of VIS/NIR Spectra and Chl-FKC

Figure 2 shows the average VIS/NIR spectra of the leaves (Figure 2(a)) and stems (Figure 2(b)) of the healthy rice plants and the plants under the stresses of Cd, Cu, DCA, and ELK.  Figure 3 shows the average Chl-FKC of the leaves (Figure 3(a)) and stems (Figure 3(b)). As shown in Figures 2 and 3, typical hyperspectral profiles of plants can be found for the leaves and stems of rice plants, as well as the Chl-FKC. Differences can be found for the leaves and stems under different stresses. However, no particular regulation (for example, which stress has higher reflectance or fluorescence intensity) could be found. There were variations during the measurement caused by the samples, instruments, and measurement conditions. Thus, it was difficult to identify the stress types by observing the differences in spectral curves or Chl-FKC. Further investigation should be conducted for stress type identification.

### 3.2. Model Establishment Using a Single Type of Feature

The full VIS/NIR spectra and Chl-FKC of leaves and stems were used as inputs of SVM and CNN-S models.  Table 3 shows the statistical results of the classification models. To construct SVM and CNN-S models, the training sets and validation sets were used, and the optimal models were selected according to the classification performances of the validation sets. For SVM, the kernel function was chosen as “rbf.” The optimization ranges of parameters *C* and *g* were both [10^−8^, 10^−7^, 10^−6^, ⋯, 10^6^, 10^7^, 10^8^]. For all deep learning models in this study, the number of epochs was set as 500. A scheduled learning rate was used by starting with 0.005 for the first 100 epochs, which was reduced to one-tenth by every 100 epochs. The batch size was set as 128. The statistical results of SVM and CNN-S models are shown in Table 3.

Both the CNN-S and SVM models performed well for L-VIS/NIR and S-VIS/NIR. CNN-S models obtained slightly better results than the corresponding SVM models, with the classification accuracy of the training, validation, and testing sets all over 90%. For L-Chl-FKC and S-Chl-FKC, both CNN-S models and SVM models failed to obtain satisfactory results, with the classification accuracy of the training, validation, and testing sets over 70%. CNN-S models and SVM models obtained close results. It could be noted that overfitting occurred for both the SVM and CNN models using chlorophyll fluorescence induction kinetic spectra. For VIS/NIR spectra, the classification models of leaves performed better than the corresponding models of stems, indicating that leaves were more suitable for rice stress type identification when using HSI. For Chl-FKC, the classification models of leaves obtained close results to those of stems.

**Figure 6:**
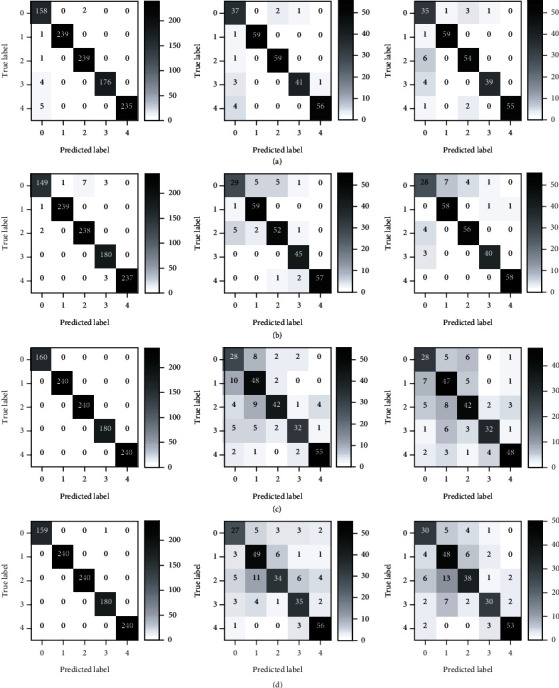
Confusion matrix of CNN-S models using VIS/NIR spectra of leaves and stems and Chl-FKC of leaves and stems, respectively. (a) The confusion matrix of the training, validation, and testing sets (from left to right) of the CNN-S model using VIS/NIR spectra of leaves. (b) The confusion matrix of the training, validation, and testing sets (from left to right) of the CNN-S model using VIS/NIR spectra of stems. (c) The confusion matrix of the training, validation, and testing sets (from left to right) of the CNN-S model using Chl-FKC of leaves. (d) The confusion matrix of the training, validation, and testing sets (from left to right) of the CNN-S model using Chl-FKC of stems.

Figure 6 shows the confusion matrix of CNN models using the four data types. No particular regulation could be found for the misclassification of samples among different classes. One sample under one type of stress could be misclassified as any other type of stress. The results of the confusion matrix showed the feasibility to identify the stress types of Cd, Cu, DCA, and ELK using HSI and Chl-FI.

### 3.3. Model Establishment Using Fused Datasets

For low-level fusion, the fused datasets were constructed according to the fusion strategies in Table 2. The fused datasets were also used as inputs of the CNN-S models with the same architectures as Figure 1. The parameter settings of the CNN-S models for fused datasets were the same as those of the CNN-S models for a single type of feature. The results of the CNN models using the fused datasets are presented in Table 4. The CNN-S model using the fusion of L-VIS/NIR and S-VIS/NIR obtained slightly better results than that using VIS/NIR spectra of leaves and stems individually. The CNN-S model using the fusion of L-Chl-FKC and S-Chl-FKC obtained significantly better results than that using features of leaves and stems individually. For fusion of VIS/NIR spectra and chlorophyll fluorescence induction kinetic spectra of leaves, the performance of the CNN model was worse than that of the CNN-S model using L-VIS/NIR and significantly better than that of the CNN-S model using L-Chl-FKC. A similar phenomenon could be found for stems. Classification models using the fusion of L-VIS/NIR and L-Chl-FKC showed better classification performances than the corresponding models using the fusion of S-VIS/NIR and S-Chl-FKC. The CNN-S model using the fusion of VIS/NIR spectra and Chl-FKC of leaves and stems showed good performances, and the performances were close to those of the CNN-S model using L-VIS/NIR.

As for the end-to-end fusion approach for middle-level fusion, the original VIS/NIR spectra and Chl-FKC of leaves and stems were fed into an end-to-end deep learning fusion model (shown in Figure 4) according to the fusion strategies in Table 2. By fusing L-VIS/NIR and S-VIS/NIR, the CNN model obtained good results, with the classification accuracy of the training, validation, and testing sets over 90%. The CNN model using the fusion of L-Chl-FKC and S-Chl-FKC obtained worse results, with the classification accuracy of the training, validation, and testing sets over 80%. When L-VIS/NIR and L-Chl-FKC were fused, the corresponding CNN model obtained good performances, with the classification of all the three sets over 90%. The classification accuracy of the training, validation, and testing sets in the CNN model using the fusion of S-VIS/NIR and S-Chl-FKC was over 80%. When VIS/NIR spectra and Chl-FKC of leaves and stems were all fused, the corresponding CNN model obtained the best performances, with the classification accuracy of all the three sets over 95%. The CNN model using the fusion of L-VIS/NIR and S-VIS/NIR obtained slightly better results than that using L-VIS/NIR and S-VIS/NIR individually. The CNN model using the fusion of L-Chl-FKC and S-Chl-FKC obtained equivalent or better results than that using L-Chl-FKC and S-Chl-FKC individually. As for the two-stage fusion approach of middle-level fusion, the deep features extracted from the VIS/NIR spectra and Chl-FKC of leaves and stems by the corresponding CNN-S models were all used for fusion. Better performances were obtained, with the classification accuracy of the training, validation, and testing sets all over 97%.

As for high-level fusion, an end-to-end CNN model for decision fusion was also applied. The classification results were the best, and the classification accuracy of the training, validation, and testing sets was 100%, 97.4%, and 97.7%. A two-stage high-level fusion approach was also used for comparison, and close results were obtained. The classification accuracy of the training, validation, and testing sets was 100%, 97.7%, and 97.7%.

The high-level fusion showed the best performances of rice stress type identification compared with low-level fusion and middle-level fusion. High-level fusion depended on the outputs of each single model. The results of the classification models using each single type of feature illustrated the potential of different types of features for rice stress type identification. The modeling performances varied for each type of feature. High-level fusion could reduce the interference by the defects of different decision models using different features, producing excellent performances. The overall results illustrated the effectiveness of high-level fusion for rice stress type identification.

Compared with end-to-end fusion models, the two-stage information fusion approaches were more complex. The features were extracted by the trained and optimized models for two-stage middle-level fusion. Moreover, one more classifier should be trained on the combined features. The whole procedure was complex, with more computation and manual intervention (here, 5 models should be trained and optimized). For two-stage high-level fusion, the predicted probability distribution vectors of the CNN-S model using each type of feature were first extracted. A decision-making procedure was implemented to make the final decision. End-to-end models for information fusion showed a simpler operation with only one-stage calculation.

As shown in Table 4, the models using the fusion of L-Chl-FKC and S-Chl-FKC obtained better results than those using L-Chl-FKC and S-Chl-FKC individually. The results indicated that combining Chl-FKC features could improve classification performances. Since the CNN-S models using L-VIS/NIR and S-VIS/NIR individually obtained good performances, the models using the fusion containing the features of L-VIS/NIR and S-VIS/NIR all obtained good performances. The results were not good when CNN-S models were built using L-Chl-FKC and S-Chl-FKC individually. The fusion of Chl-FKC features and the corresponding VIS/NIR features showed better results than the models using the corresponding Chl-FKC features and obtained lower or close results than the models using the corresponding VIS/NIR features. Due to the gap between the performances of models using VIS/NIR spectra and the corresponding Chl-FKC individually, the fusion of Chl-FKC features and the corresponding VIS/NIR features obtained the performances between the performances of the models using each type of feature individually.

The fusion of all the features showed relatively better performances. By fusing all the types of features, the complementary information relating to rice stress type identification hidden in the obtained features could be revealed for rice stress type identification.

**Figure 7:**
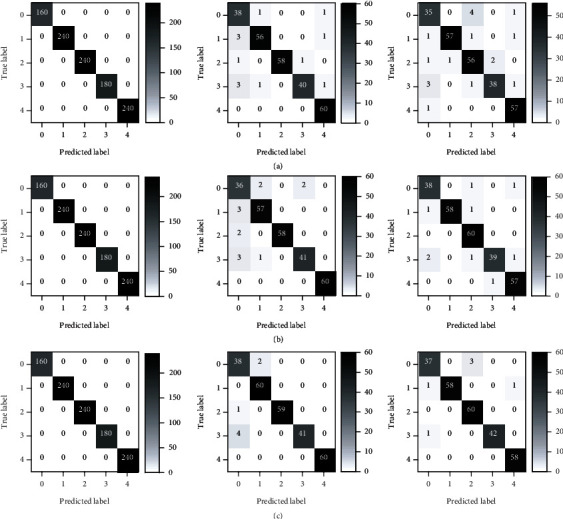
Confusion matrices of the three levels of end-to-end information fusion using the Fusion 5 strategy. (a) The confusion matrix of the training, validation, and testing sets (from left to right) of low-level fusion. (b) The confusion matrix of the training, validation, and testing sets (from left to right) of middle-level fusion. (c) The confusion matrix of the training, validation, and testing sets (from left to right) of high-level fusion.

Furthermore, the confusion matrices of the three levels of information fusion using the Fusion 5 strategy were explored (shown in Figure 7). Although Cd and Cu were both heavy metal stresses and DCA and ELK were herbicide stresses, no particular rules could be found for the misclassification of samples among different classes. The good performances of the classification of rice plants under different stress types showed that information fusion of phenotyping traits of leaves and stems acquired by HSI and Chl-FI had great potential for rice stress type identification.

## 4. Discussion

The effectiveness of HSI and Chl-FI for high-throughput plant stress phenotyping has been widely verified in various studies [10, 13, 56–60]. In this study, VIS/NIR spectra acquired by HSI and chlorophyll fluorescence induction kinetic spectra acquired by Chl-FI of leaves and stems of rice plants were used to identify the stress types, and the results were promising.

In previous studies, SVM has been widely used as a conventional machine learning method to compare with deep learning methods for 1D spectral analysis. The results between SVM and deep learning methods were quite close [61–64]. This study found similar trends for plant stress type identification using VIS/NIR spectra and chlorophyll fluorescence induction kinetic spectra of leaves and stems. The 1D VIS/NIR spectra and chlorophyll fluorescence induction kinetic spectra have simple data structures, and the potential of CNN models for deep feature learning could not be fully revealed. On the other hand, the number of samples was not large enough, which were suitable for SVM. The potential of CNN models for big data could not be fully revealed.

Moreover, variations can be found between VIS/NIR spectra and chlorophyll fluorescence induction kinetic spectra for stress type identification. As for HSI, the classification performances of CNN models showed slightly better results than those of SVM models. As for Chl-FI, the classification performances of CNN models using Chl-FKC showed close results to those of SVM models. Models using VIS/NIR spectra performed better than those using chlorophyll fluorescence induction kinetic spectra. In some other studies, the performances for plant phenotyping using features of HSI were better than those using features of Chl-FI [18, 22, 65]. Although spectral indices calculated from VIS/NIR spectra are widely used for analysis, the full-range spectra are also widely used for analysis. Unlike VIS/NIR spectra, Chl-FI parameters calculated from Chl-FKC were more widely used for analysis in Chl-FI rather than the full-range Chl-FKC. The results illustrated the potential of stress type identification using Chl-FKC, and more efforts should be made to improve the performances. VIS/NIR can reflect the physiological and biochemical changes under different stresses, and Chl-FI is an efficient technique to assess the status of plant photosynthesis. VIS/NIR spectra may provide more information than Chl-FKC.

Both leaves and stems showed promising results for plant stress type identification, and the performances of leaves were better than those of stems. In general, leaves were more likely to be used for plant phenotyping. Fewer studies have studied the high-throughput phenotyping of stems [66–73]. The overall results showed that stems can be an efficient alternative for plant phenotyping in addition to leaves. Stems and leaves showed different phenotyping traits under different stresses. The good performances indicated that the combinations of leaves and stems have the great potential to provide more information for plant stress phenotyping.

HSI and Chl-FI acquired different phenotyping traits of leaves and stems. The fusion of VIS/NIR spectral features and Chl-FKC features combined different aspects of features of plants. The results showed that using the fusion of VIS/NIR spectral features and Chl-FKC features had the potential to improve the performances of plant stress type identification. In some other studies, the authors also fused data from different sensors to improve the performances of plant phenotyping [30, 74, 75].

With the advantage of deep learning on feature learning and classification tasks, end-to-end CNN models were designed to implement the information fusion of phenotyping traits acquired from leaves and stems by HSI and Chl-FI. The most widely used two-stage information fusion consisted of training or manually selecting features, concatenating features manually, and training an independent classifier using fused features. The end-to-end deep fusion models combined all these steps into one model, and the whole procedure was implemented using one-stage training. These models were much simpler and did not need manual intervention to extract or select appropriate features. By obtaining the phenotyping traits, these phenotyping traits could be directly fed into the end-to-end deep fusion models, which had great potential for real-world application.

With the development of advanced phenotyping techniques, various aspects of phenotyping traits can be obtained. How to fully reveal the information relating to plant growth status from various aspects of phenotyping traits is of importance. Information fusion provided an effective alternative to combine different aspects of phenotyping traits to reveal the phenotyping information related to plant growth status fully. The effectiveness of information fusion for rice stress type identification proved that information fusion was promising for plant growth status evaluation. On the other hand, different organs have different phenotyping traits, even measured by the same techniques. Thus, different organs could be analyzed to evaluate the plant growth status. The combination of phenotyping traits of different organs will provide more information of plants, and how to effectively fuse the phenotyping traits of different organs remains a challenging issue. This study provided an efficient alternative for the fusion of the phenotyping traits of different organs for plant phenotyping, which could enhance the high-throughput phenotyping performances, including the abiotic stresses caused by environmental factors.

## Figures and Tables

**Table 1:** Descriptions of the dataset.

Data source	Number of features	Number of samples		
		Training	Validation	Testing
L-VIS/NIR	396	1060	265	261
S-VIS/NIR	396	1060	265	261
L-Chl-FKC	286	1060	265	261
S-Chl-FKC	286	1060	265	261

**Table 2:** The fusion strategies used in this study.

Fusion strategy	L-VIS/NIR	S-VIS/NIR	L-Chl-FKC	S-Chl-FKC
Fusion 1	✓^‡^	✓	×	×
Fusion 2	×	×	✓	✓
Fusion 3	✓	×	✓	×
Fusion 4	×	✓	×	✓
Fusion 5	✓	✓	✓	✓

‡The symbol ✓ means that the corresponding features were used for fusion, and the symbol × means that the corresponding features were not used for fusion.

**Table 3:** The classification results of SVM and CNN models using L-VIS/NIR, S-VIS/NIR, L-Chl-FKC, and S-Chl-FKC.

Dataset type	Model	Accuracy (%)		
		Training	Validation	Testing
L-VIS/NIR	SVM	100	91.7	92.0
	CNN	98.8	95.1	92.7
S-VIS/NIR	SVM	96.2	87.9	94.7
	CNN	98.4	91.3	92.0
L-Chl-FKC	SVM	100	74.3	75.5
	CNN	100	77.4	75.5
S-Chl-FKC	SVM	92.9	78.1	78.9
	CNN	99.9	75.8	76.2

**Table 4:** The classification results of the CNN models of each fusion level.

Fusion level	Fusion strategy	Accuracy (%)		
		Training	Validation	Testing
Low level	Fusion 1	99.9	97.4	94.6
	Fusion 2	100	79.2	80.5
	Fusion 3	100	91.7	89.3
	Fusion 4	100	85.3	85.8
	Fusion 5	100	95.1	93.1
	Fusion 5-TwoStage^§^	/	/	/

Middle level	Fusion 1	97.6	95.1	92.3
	Fusion 2	99.9	84.5	82.8
	Fusion 3	100.0	95.5	91.6
	Fusion 4	99.6	84.9	85.4
	Fusion 5	100	95.1	96.6
	Fusion 5-TwoStage^||^	100	97.4	97.3

High level	Fusion 1	99.6	95.5	95.4
	Fusion 2	100	94.3	93.5
	Fusion 3	99.5	89.1	87.0
	Fusion 4	100	86.4	85.1
	Fusion 5	100	97.4	97.7
	Fusion 5-TwoStage^¶^	100	97.7	97.7

§Low-level fusion directly fuses the raw data as the input of the model, which does not need a two-stage training. ||The two-stage-based middle-level fusion model fuses the deep features extracted by single models (CNN-S). Another CNN classifier is trained for processing the fused features. ¶The two-stage-based high-level fusion model fused 4 predicted probability distribution vectors (each includes 5 elements). The averaged probability distribution vectors are for making the final decision.

## Data Availability

The data used in this study are available from the corresponding author upon reasonable request.
